# Beyond Aquaporins: Tamoxifen Preserves Collecting Duct Epithelial Homeostasis in Lithium-Induced Nephrogenic Diabetes Insipidus

**DOI:** 10.3390/cells15211953

**Published:** 2026-10-27

**Authors:** Mariangela Centrone, Ines Angelini, Mariagrazia D’Agostino, Annarita Di Mise, Barbara Barile, Grazia Paola Nicchia, Giampaolo Morciano, Stine Julie Tingskov, Xabier Sørtvedt, Birgitte Mønster Christensen, Marianna Ranieri, Giovanna Valenti, Rikke Nørregaard, Grazia Tamma

**Affiliations:** 1 Department of Biosciences Biotechnologies and Environment, University of Bari “Aldo Moro”, 70125 Bari, Italy; ines.angelini@uniba.it (I.A.); mariagrazia.dagostino@uniba.it (M.D.); barbara.barile@uniba.it (B.B.); graziapaola.nicchia@uniba.it (G.P.N.); giampaolo.morciano@uniba.it (G.M.); marianna.ranieri@uniba.it (M.R.); giovanna.valenti@uniba.it (G.V.); 2 Department of Biotechnology and Biosciences, University of Milano-Bicocca, 20126 Milan, Italy; annarita.dimise@unimib.it; 3 Department of Clinical Medicine, Aarhus University, 8200 Aarhus N, Denmark; sjt@biomed.au.dk (S.J.T.); rn@clin.au.dk (R.N.); 4 Department of Biomedicine, Aarhus University, 8000 Aarhus C, Denmark; xs@biomed.au.dk (X.S.); bmc@biomed.au.dk (B.M.C.); 5 Department of Renal Medicine, Aarhus University Hospital, 8200 Aarhus N, Denmark

**Keywords:** lithium, necroptosis, lithium-induced nephrotoxicity, nephrogenic diabetes insipidus (NDI), tamoxifen, aquaporins, primary cilium, EMT

## Abstract

Lithium is widely used to treat psychiatric disorders, including bipolar disorder. However, long-term lithium therapy is frequently associated with acquired nephrogenic diabetes insipidus (NDI), a disorder characterized by impaired urinary concentrating ability and excessive water loss. Tamoxifen (TAM), a selective estrogen receptor modulator, has previously been shown to alleviate lithium-induced NDI by restoring AQP2 and AQP3 expression. Here, we investigated whether necroptosis-related signaling, primary cilium remodeling, and epithelial–mesenchymal transition (EMT)-like changes contribute to lithium-induced collecting duct alterations and whether TAM modulates these responses. Using complementary in vitro, ex vivo, and in vivo approaches, we found that lithium significantly reduced the expression of the necroptosis-related proteins receptor-interacting protein kinase 3 (RIPK3) and mixed lineage kinase domain-like protein (MLKL), whereas TAM prevented their downregulation. Lithium also induced primary cilium elongation and promoted EMT-like changes, characterized by reduced E-cadherin and increased vimentin expression, which were attenuated by TAM. Collectively, these findings demonstrate that lithium profoundly alters collecting duct epithelial homeostasis by modulating the abundance of necroptosis-related proteins, primary cilium morphology, and epithelial phenotype. The ability of TAM to counteract these alterations indicates that its protective effects extend beyond aquaporin regulation, supporting its potential as a therapeutic strategy for lithium-induced NDI.

## 1. Introduction

Lithium treatment is approved for bipolar disorder and is also used for depressive disturbs [1], as it is a good mood stabilizer and reduces suicidal tendencies in patients with mental illness [2]. However, lithium therapy is known to cause chronic renal toxicity, which can also lead to acquired nephrogenic diabetes insipidus (NDI) [3,4,5]. Immunoblotting and immunoelectron microscopy experiments showed that lithium significantly reduced the expression of AQP2 and AQP3 water channels, whose reduction is associated with polyuria [5] and the onset of NDI. The expression of AQP2 is tightly regulated and induced by different transcription factors. A recent study integrating multiple-omics dataset using a Bayesian network approach identified several key regulators of AQP2 expression, including Elf3, Cebpb, Atf1 [6]. Treatment with tamoxifen (TAM), a selective estrogen receptor modulator, mitigates the lithium-induced changes in renal sodium and water transporters and channels [7]. Specifically, TAM counteracted the lithium-induced increase in urinary sodium excretion by modulating the expression of βENaC and γENaC [7] and prevented the development of the lithium-induced NDI by restoring the expression levels of AQP2 and AQP3 [8,9]. Loss of AQP2 in lithium-induced NDI may reflect broader alterations in collecting duct cell homeostasis, involving dysregulated autophagy, apoptosis, cell proliferation, oxidative stress, inflammation, and epithelial remodeling, as suggested by integrated transcriptomic and proteomic analyses [10]. Recent studies indicate that renal epithelial remodeling may involve the activation of epithelial plasticity programs displaying features of epithelial–mesenchymal transition (EMT) [11]. These processes are characterized by changes in epithelial differentiation, cytoskeletal organization, and cell fate, ultimately contributing to tissue remodeling and adaptation to injury. Whether EMT-related mechanisms contribute to lithium-induced collecting duct remodeling remains largely unexplored. A significant reduction in the protein inhibitor of cyclin-dependent kinases p27 (KIP), consistent with an increase in cellular proliferation and remodeling, was observed in the renal medulla of lithium-treated animals by cDNA microarray screening [12]. Interestingly, four weeks treatment with lithium markedly reduced AQP2 protein expression and significantly decreased the proportion of principal cells while increasing the proportion of intercalated (H^+^-ATPase-positive) cells [13]. Consistent with these findings, time-course experiments in rats showed that over 4 days, lithium significantly increased the proliferation of renal cells double-positive stained with AQP4 and PCNA, which are principal cell and proliferation biomarkers, respectively. However, over 10–15 days, there was also a marked increase in the proliferation of intercalated cells that stained positive for H^+^-ATPases [14]. These effects strongly suggest that lithium induces a dramatic remodeling of tubular cell composition. Accordingly, lithium-induced downregulation of Notch signaling promotes the transdifferentiation of mature principal cells into intercalated cells in the adult kidney. This may be due to the inactivation of HES1, which is primarily involved in the activation of Foxi1, the key transcription factor responsible for controlling the intercalated cell program [15,16]. Thus, changes in the composition of tubular cells could be correlated with dynamic modulation of cell fate programs. More recently, metabolomic, transcriptomic, and proteomic studies have shown that lithium treatment induces relevant metabolic reprogramming leading to the stimulation of MAPK, mTOR, and Akt pathways that control cell survival and apoptotic pathways [17,18,19]. Furthermore, lithium also induces impairment of focal adhesion and actin cytoskeletal remodeling [18,19] by modulating the expression and the phosphorylation level of proteins involved in controlling actin dynamics [18,20]. Given the profound structural and phenotypic remodeling induced by lithium in collecting duct cells, we hypothesized that lithium may also modulate necroptosis-related signaling as part of the cellular remodeling process associated with lithium-induced NDI. The present study, therefore, investigated the effect of lithium on the cell necroptosis-related pathway. Necroptosis is an inflammatory programmed cell death that is modulated by the receptor-interacting protein kinases 1 and 3 (RIPK1 and RIPK3) and the mixed lineage kinase domain-like protein (MLKL). When stimulated by death receptors, RIPK1 forms a complex with RIPK3, which phosphorylates MLKL, causing the cell to swell and eventually die [21]. Here, we demonstrated that treatment with lithium downregulated the abundance of necroptosis cell program markers and increased the primary cilium length. Importantly, these lithium-induced responses were prevented by TAM administration.

## 2. Materials and Methods

### 2.1. Chemicals and Antibodies

Cell culture media and FBS (fetal bovine serum) were obtained from GIBCO (Thermo Fisher Scientific^TM^, Waltham, MA, USA). Lithium Chloride (Cat. L4408), monoclonal antibody against Acetyl-Alpha-Tubulin (Lys40, Cat. 66200-1-Ig, dilution 1:1000), and rabbit polyclonal antibody against E-cadherin (Cat. 07-697, dilution 1:800) were purchased from Merck (Merck KGaA, Darmstadt, Germany). 4-Hydroxytamoxifen (Cat. HY-16950) was obtained by MedChemExpress (D.B.A. Italia S.r.l. Segrate, Milan, Italy). Polyclonal antibody against Caspase-3 (Cat. PA5-77887; dilution 1:1000) was obtained from Invitrogen (Thermo Fisher Scientific, Waltham, MA, USA). Rabbit polyclonal antibody against vimentin (Cat. 10366-1-AP; dilution 1:2500) was purchased from Proteintech (Proteintech Group, Inc., Rosemont, IL, USA). Monoclonal antibody against RIP3 (sc-374639; dilution 1:2000) and monoclonal antibody against PCNA (PC10, Cat. sc-56; dilution 1:200) were purchased from Santa Cruz Biotechnology (Tebu Bio, Milan, Italy). Polyclonal antibody against MLKL (Cat. orb536567, dilution 1:500) was obtained from Biorbyt (D.B.A. Italia S.r.l. Segrate, Milan, Italy). Monoclonal antibody against LC3A/B (D3U4C, Cat. 12741; dilution 1:1000) and antibody against cleaved Caspase-3 (Cat. 9662; dilution 1:1000) were obtained from Cell Signaling Technology (Danvers, MA, USA). E-cadherin immunohistochemistry was performed using rabbit polyclonal anti-E-cadherin antibody (Cat. PA5-85088; dilution of 1:500) and obtained from Invitrogen. Anti-rabbit horseradish peroxidase-conjugated secondary antibody (Cat. P0448; dilution 1:200) was purchased from DAKO, Glostrup, Denmark. The anti-Rabbit IgG and the anti-Mouse IgG both conjugated to horseradish peroxidase (HRP) were obtained from Sigma-Aldrich (Merck KGaA, Darmstadt, Germany). Secondary goat anti-rabbit Alexa488 conjugated antibodies were from Molecular Probes (Thermo Fisher Scientific, Waltham, MA, USA).

### 2.2. Cell Culture and Treatment

Mouse Cortical Collecting Duct cells (MCD4), stably expressing human AQP2 and V2R and described by Angelini et al. [22], were grown in Dulbecco’s modified Eagle’s medium (DMEM/F12) supplemented with 5% (*v*/*v*) fetal bovine serum, 1% (*v*/*v*) L-glutamine, 100 IU/mL penicillin, 100 μg/mL streptomycin, 5 µM dexamethasone, 400 µg/mL G418 (for AQP2 resistance) and 1 µg/mL puromycin (for V2R resistance) at 37 °C in 5% CO_2_.

Cells were left under basal conditions (Untreated), treated with 1 or 10 mM lithium chloride (LiCl) for 48 h and/or treated with 2 µM 4-Hydroxytamoxifen (TAM).

### 2.3. Cell Lysates

MCD4 cells were seeded onto 6-well plates and treated as mentioned before. Then cells were homogenized as previously described [23] in an ice-cold RIPA buffer (150 mM NaCl, 10 mM Tris-HCl pH 7.2, 0.1% SDS, 1.0% Triton X-100, 1% Na deoxycholate and 5 mM EDTA pH 7.5), in the presence of proteases (1 mM PMSF, 2 mg/mL leupeptin, and 2 mg/mL pepstatin A) and phosphatases (10 mM NaF and 1 mM sodium orthovanadate) inhibitors. Suspensions were then centrifuged at 12,000× *g* for 10 min at 4 °C, and supernatants were used for Western blotting analysis.

### 2.4. Kidney Slices from Mouse Inner Medullary Collecting Ducts

Mice were anesthetized and euthanized by dislocation. Kidneys were quickly removed and ~500 µm sections were made as previously described [24]; only the inner medulla was used for the experiments. Obtained kidney inner medulla slices were equilibrated for 10 min in a buffer containing 118 mM NaCl, 16 mM Hepes, 17 mM Na-Hepes, 14 mM glucose, 3.2 mM KCl, 2.5 mM CaCl_2_, 1.8 mM MgSO_4_, and 1.8 mM KH_2_PO_4_ (pH 7.4). Subsequently, kidney slices were left under basal conditions or incubated with 100 mM lithium chloride (LiCl) and/or 20 µM 4-Hydroxytamoxifen (TAM) for 4 h at 37 °C in 5% CO_2_. Treated sections were minced with scissors in the same buffer in the presence of protease and phosphatase inhibitors, sonicated (80 Hz for 20 s) and centrifuged at 12,000× *g* for 10 min at 4 °C. Supernatants were collected and used for Western blotting analysis.

### 2.5. Animals (In Vivo Study)

All procedures were conducted in accordance with the Danish Animal Welfare Act and carried out in collaboration with a licensed veterinarian. Experimental protocols were approved by both the local and national ethical review committee (Danish Animal Experiments Inspectorate) and performed under the authority of the Project License (no. 2015-15-0201-00658).

For the NDI study, adult male Sprague-Dawley rats (initial body weight: 198 ± 11.5 g) were assigned to four groups: 1) control rats receiving standard chow for 14 days (*n* = 9 for WB and *n* = 2 for IHC), (2) LiCl rats receiving chow supplemented with lithium chloride (40 mmol/kg dry food) for 14 days (*n* = 9 for WB and *n* = 4 for IHC), and 4) LiCl + TAM50 rats receiving LiCl for 14 days and tamoxifen (50 mg/kg) during the final 7 days (*n* = 9 for WB and *n* = 6 for IHC). Lithium-treated rats were randomized to the three LiCl groups after 7 days of LiCl administration. TAM (Merck KGaA, Darmstadt, Germany; Co. T5648) was dissolved in ethanol (EtOH) and mixed with corn oil (Merck KGaA, Darmstadt, Germany). TAM was administered daily via oral gavage. After 14 days, the rats were sacrificed, and the kidneys were collected for Western blotting (WB) and immunohistochemistry (IHC). This same cohort of rats was used in our previously published studies investigating the effects of TAM on AQP2 and AQP3 regulation, as well as its role in natriuresis in NDI rats [7,8,9].

### 2.6. Gel Electrophoresis and Immunoblotting 

Proteins were separated by SDS-PAGE using stain-free polyacrylamide gels (Bio-Rad Laboratories, Inc., Hercules, CA, USA). Protein bands were electrophoretically transferred onto Immobilon-P membranes (Millipore Corporate Headquarters, Billerica, MA, USA), blocked in EveryBlot Blocking Buffer (Bio-Rad Laboratories, Milan, Italy), and incubated with primary antibodies overnight. Immunoreactive bands were detected with a secondary antibody conjugated to horseradish peroxidase (HRP). Membranes were developed using Clarity Western ECL Substrate with the ChemiDoc System gels (Bio-Rad Laboratories, Milan, Italy). Obtained bands were normalized to total protein using stain-free technology gels. Densitometry was performed using Image Lab software 6.1 (Bio-Rad Laboratories, Milan, Italy).

### 2.7. Immunofluorescence Staining

Confocal studies were performed as previously described [22]. MCD4 cells were grown on cell culture polyethylene terephthalate (PET) inserts, and at complete confluence, cells were exposed to a serum-starved medium for 48 h and were left untreated or stimulated as mentioned before. Then, cells were fixed in 4% paraformaldehyde, washed in phosphate-buffered saline (PBS), and permeabilized with 0.5% Triton X-100 in PBS. After blocking with 1% bovine serum albumin in PBS for 30 min, cells were incubated with anti-Acetyl-α-Tubulin primary antibody overnight at 4 °C. After rinsing, cells were incubated for 1 h with the appropriate Alexa Fluor-conjugated secondary antibody, followed by DAPI for 10 min. After washing, samples were mounted onto glass slides with Mowiol (Merck KGaA, Darmstadt, Germany). Confocal Z-stack images were acquired with Nikon V3 Spinning Disk confocal microscope by using Plan Apo 60×/1.40 NA oil and cilium length was analyzed with NIS-Elements Advanced Research software 6.01.00. Briefly, xz confocal image stacks were reconstructed as maximum intensity projection images prior to length measurement by tracing the ciliary shaft with the line measurement tool. A minimum of three independent experiments were analyzed.

### 2.8. Immunohistochemistry on Kidney Tissue

Paraffin-embedded sections (2 µm) were rehydrated and blocked with 0.3% H_2_O_2_ in methanol for 30 min followed by antigen retrieval in TEG-buffer. The sections were treated with 50 mM NH_4_Cl in PBS for 30 min followed by washing with 1% bovine serum albumin (BSA), 0.2% gelatin, 0.05% saponin in PBS. The sections were incubated with either anti-E-cadherin antibody (Cat. PA5-85088, Invitrogen, Thermo Fisher Scientific, Waltham, MA, USA, dilution 1:500), or anti-vimentin (Cat. 10366-1-AP, Proteintech Group, Inc., Rosemont, IL, USA, dilution 1:400) overnight at 4 °C. Next day, sections were rinsed in 0.1% BSA, 0.2% gelatin, 0.05% saponin in PBS, and incubated with corresponding anti-rabbit horseradish peroxidase-conjugated secondary antibody (P0448 at dilution 1:200, DAKO, Glostrup, Denmark) for 1 h. Labeling was detected with 3,3′-Diaminobenzidine tetrahydrochloride (DAB) and sections were counterstained with Mayers Hematoxylin. Sections were analyzed with a Leica DM2500 microscope (Leica Microsystems GmbH, Wetzlar, Germany) and images were acquired with a mounted MC170 HD camera (Leica Microsystems GmbH, Wetzlar, Germany).

### 2.9. Statistical Analysis

All values are reported as means ± S.E.M. Statistical analysis was performed by one-way ANOVA, followed Tukey for multiple comparisons test. The difference of *p* < 0.05 was considered statistically significant.

## 3. Results

### 3.1. In Vivo and Ex Vivo Action of Lithium on Necroptosis Pathway Markers

**Figure 1:**
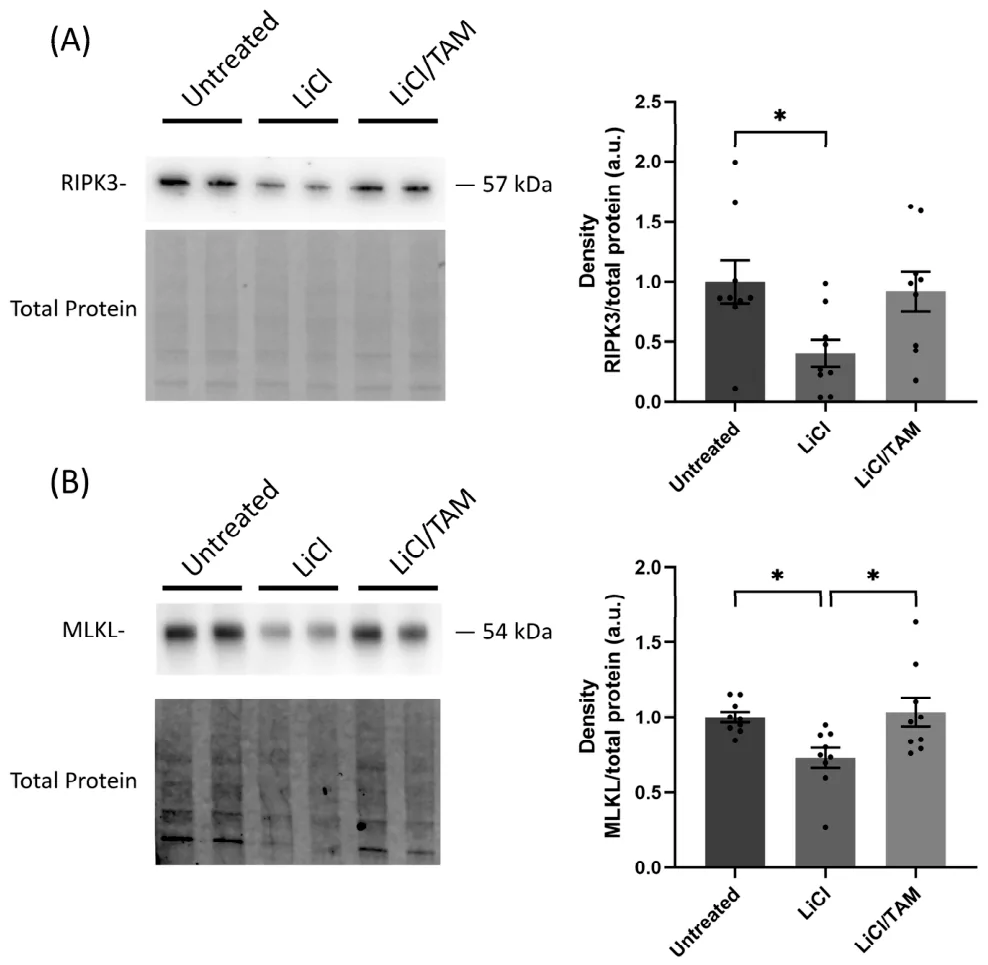
Effects of lithium on RIPK3 and MLKL expression in rats with lithium-induced NDI (in vivo study). An equal amount (60 µg) of proteins from rat kidney inner medulla lysates was blotted and probed with the indicated antibodies. Immunodetection revealed that RIPK3 (**A**) and MLKL (**B**) protein expression is significantly decreased in rats treated with lithium compared to control rats (Untreated). Of note, treatment with TAM prevented the lithium-induced decrease in both RIPK3 and MLKL (LiCl/TAM). Representative immunoblots are shown. Bar graphs represent densitometric analysis from all independent experiments. Data are presented as mean ± SEM and analyzed by one-way ANOVA followed by Tukey’s multiple comparisons test (* *p* < 0.05).

In vivo experiments were carried out to evaluate the effect of lithium on the abundances of necroptosis biomarkers. Western blotting analysis was performed on kidney inner medulla lysates from rats treated with LiCl as described under methods. Compared to lysates isolated from untreated animals, a significant decrease in the protein abundances of RIPK3 (Untreated: 1.00 ± 0.18, *n* = 9 vs. LiCl: 0.40 ± 0.11, *n* = 9) and MLKL (Untreated: 1.00 ± 0.04, *n* = 9 vs. LiCl: 0.73 ± 0.07, *n* = 9) was observed in the lysates obtained from lithium-treated rats (Figure 1). Interestingly, treatment with TAM rescued the lithium-induced decrease in RIPK3 (LiCl/TAM: 0.92 ± 0.17, *n* = 9), although this effect did not reach statistical significance, whereas TAM significantly restored MLKL levels (LiCl/TAM: 1.03 ± 0.1, *n* = 9).

**Figure 2:**
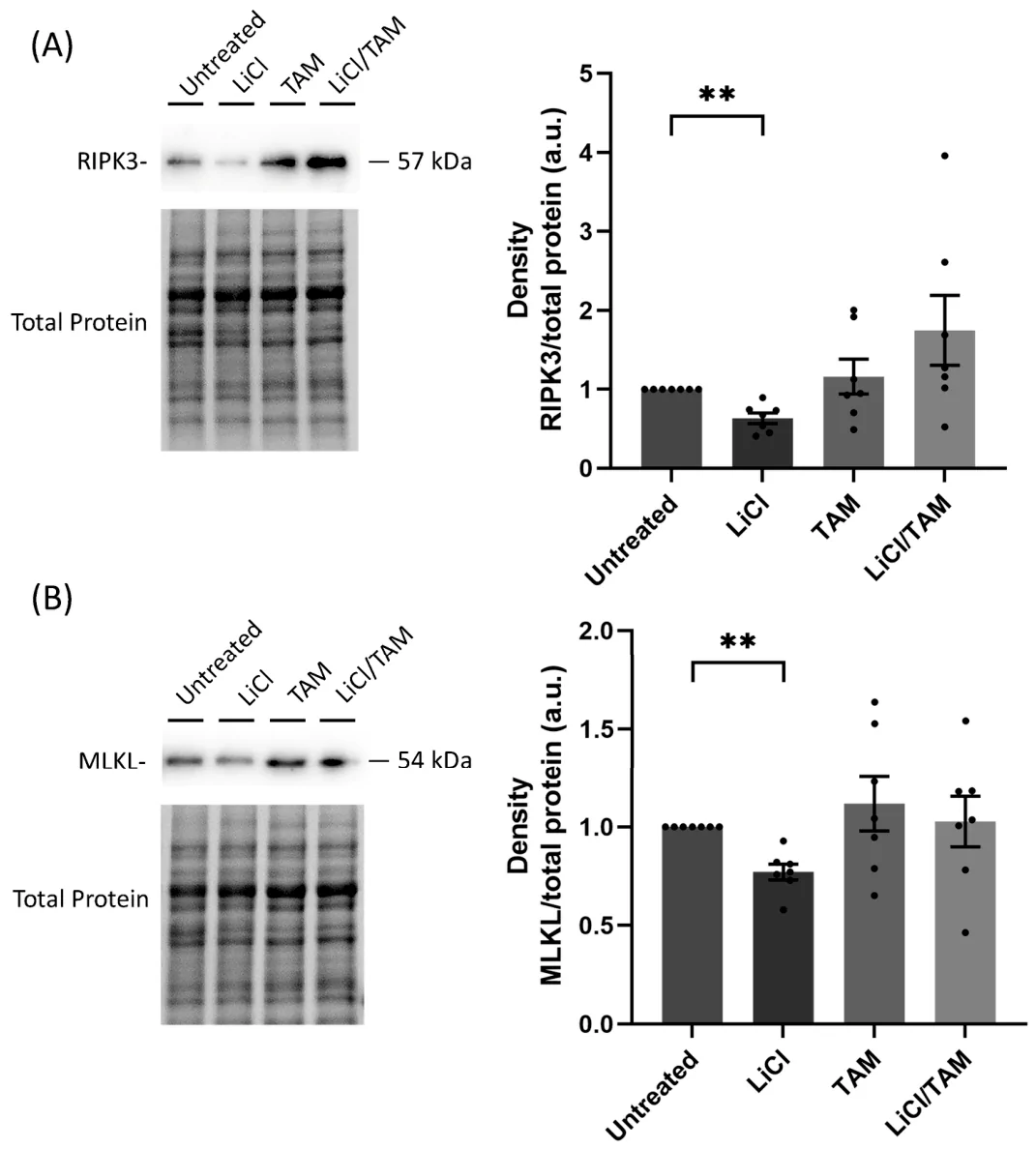
Effects of lithium on RIPK3 and MLKL expression in mouse kidney slices (ex-vivo study). Fresh mouse kidney slices were treated as described in the Materials and Methods section, and an equal amount (60 µg) of protein lysates was blotted and probed with the indicated antibodies. Immunodetection revealed that RIPK3 (**A**) and MLKL (**B**) protein expressions are significantly decreased in mouse kidney slices incubated with lithium (LiCl). Of note, this effect was prevented by co-treatment with lithium and TAM (LiCl/TAM). Incubation with TAM alone did not alter the protein abundance (TAM). Representative immunoblots are shown. Bar graphs represent densitometric analysis from all independent experiments. Data are presented as mean ± SEM and analyzed by one-way ANOVA followed by Tukey’s multiple comparisons test (** *p* < 0.01).

Importantly, these findings were confirmed in ex vivo experiments. Briefly, fresh mouse kidney slices were incubated with lithium (100 mM for 4 h) in the absence or the presence of TAM (20 µM for 4 h). Subsequent immunoblotting (Figure 2) revealed that, compared to lysates from untreated kidney slices, incubation with lithium significantly reduced the expression level of RIPK3 (Untreated: 1.00, *n* = 7 vs. LiCl: 0.63 ± 0.07, *n* = 7) and MLKL (Untreated: 1.00, *n* = 7 vs. LiCl: 0.77 ± 0.04, *n* = 7). This effect was restored by co-treatment with lithium and TAM (RIPK3 = LiCl/TAM: 1.75 ± 0.44, *n* = 7; MLKL = LiCl/TAM: 1.03 ± 0.13, *n* = 7). Incubation with TAM alone did not alter the protein abundance of RIPK3 and MLKL compared to untreated kidney slice specimens (RIPK3 = TAM: 1.16 ± 0.22, *n* = 7; MLKL = TAM: 1.12 ± 0.14, *n* = 7).

### 3.2. In Vitro Action of Lithium on Necroptosis

**Figure 3:**
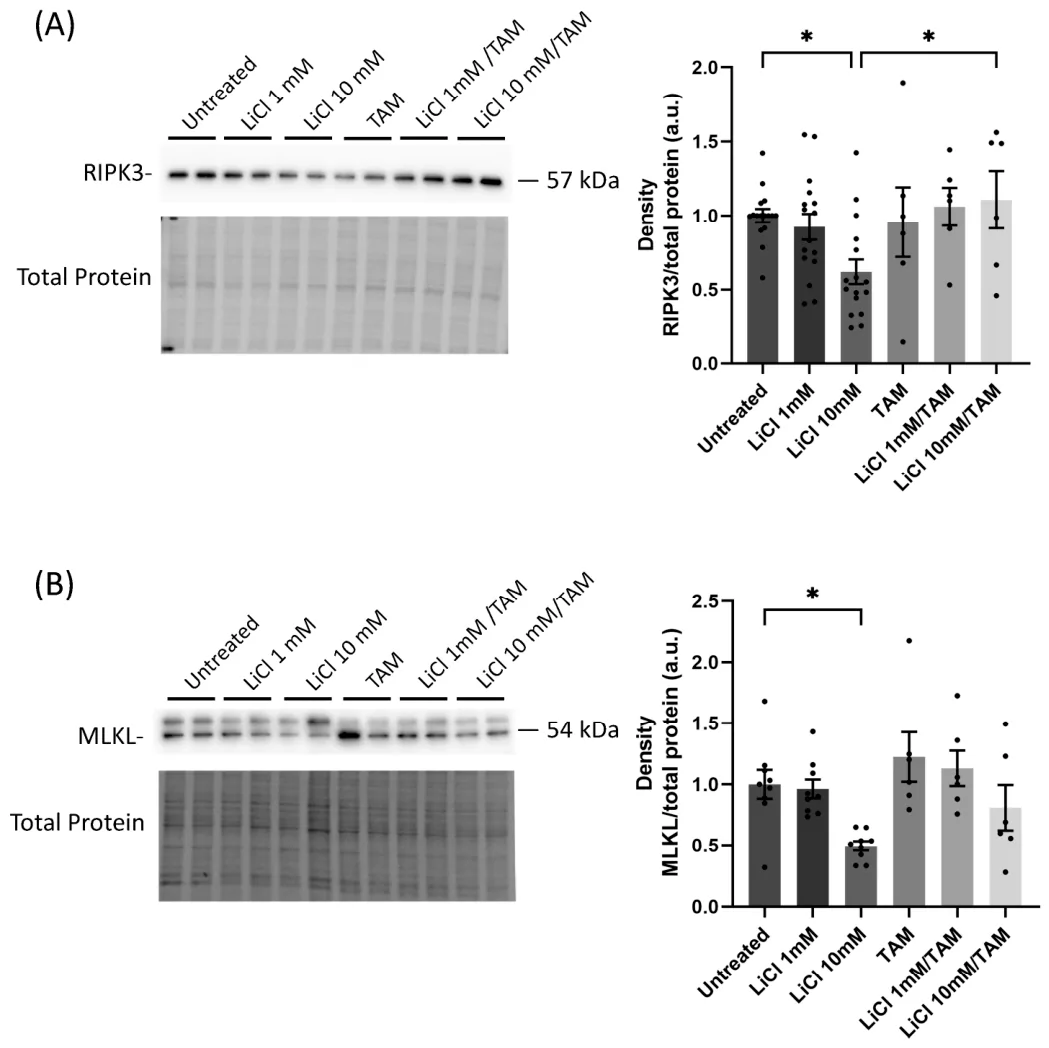
Effects of lithium on RIPK3 and MLKL expression in MCD4 cells (in vitro study). MCD4 cells were treated as described in the Materials and Methods section and an equal amount (60 µg) of cell lysates were blotted and probed with the indicated antibodies. Immunodetection revealed that RIPK3 (**A**) and MLKL (**B**) protein expression is significantly decreased in MCD4 cells incubated with lithium at 10 mM (LiCl 10 mM). This effect was prevented by co-treatment with lithium and TAM (LiCl 10 mM/TAM). Of note, treatment with lithium at the lower concentration (1 mM) did not alter the protein abundance of RIPK3 and MLKL compared to untreated cells (LiCl 1 mM). Representative immunoblots are shown. Data are presented as mean ± SEM and analyzed by one-way ANOVA followed by Tukey’s multiple comparisons test (* *p* < 0.05).

To further dissect the intracellular pathway modulated by lithium treatment, renal collecting duct MCD4 cells were used as an experimental cell model [22]. In line with the in vivo and ex vivo data, treatment with lithium (10 mM for 48 h) significantly reduced the protein expression of RIPK3 (Untreated: 1.00 ± 0.04, *n* = 16 vs. LiCl 10 mM: 0.62 ± 0.08, *n* = 16) and MLKL (Untreated: 1.00 ± 0.12, *n* = 9 vs. LiCl 10 mM: 0.49 ± 0.04, *n* = 9). The lithium-induced decrease on the RIPK3 was abolished by treatment with TAM (LiCl 10 mM/TAM: 1.11 ± 0.20, *n* = 6). A slight rescue of MLKL expression was detected under TAM treatment (LiCl 10 mM/TAM: 0.81 ± 0.19, *n* = 6). Conversely, incubation with lithium at 1 mM concentration did not alter the protein abundance of RIPK3 and MLKL compared to untreated cells (RIPK3 = LiCl 1 mM: 1.06 ± 0.13, *n* = 6; MLKL = LiCl 1 mM: 1.13 ± 0.15, *n* = 6) (Figure 3).

To further characterize the cellular responses associated with lithium exposure, the expression of markers involved in proliferation, autophagy, and apoptosis was evaluated by immunoblotting. Lithium treatment significantly increased the abundance of proliferating cell nuclear antigen (PCNA), indicating enhanced proliferative activity of collecting duct MCD4 cells. However, a similar increase was observed in cells exposed to lithium in combination with tamoxifen, with the highest PCNA levels detected in the Li 10 mM/TAM group (Figure S1). By contrast, analysis of the autophagy marker LC3 revealed no significant differences in the LC3-II/I expression among the experimental groups, suggesting that neither lithium nor tamoxifen markedly affected autophagic activity or flux under these conditions in this cell model (Figure S2). Moreover, lithium treatment did not alter the expression level of caspase-3. However, TAM treatment increased caspase-3 abundance both alone and in combination with lithium (Figure S3A). Nevertheless, no detectable increase in cleaved caspase-3 was observed under treatment with TAM. Together, these findings likely indicate that lithium and/or TAM did not alter cell apoptosis (Figure S3B).

### 3.3. In Vitro Lithium Effects on Cilium Length

**Figure 4:**
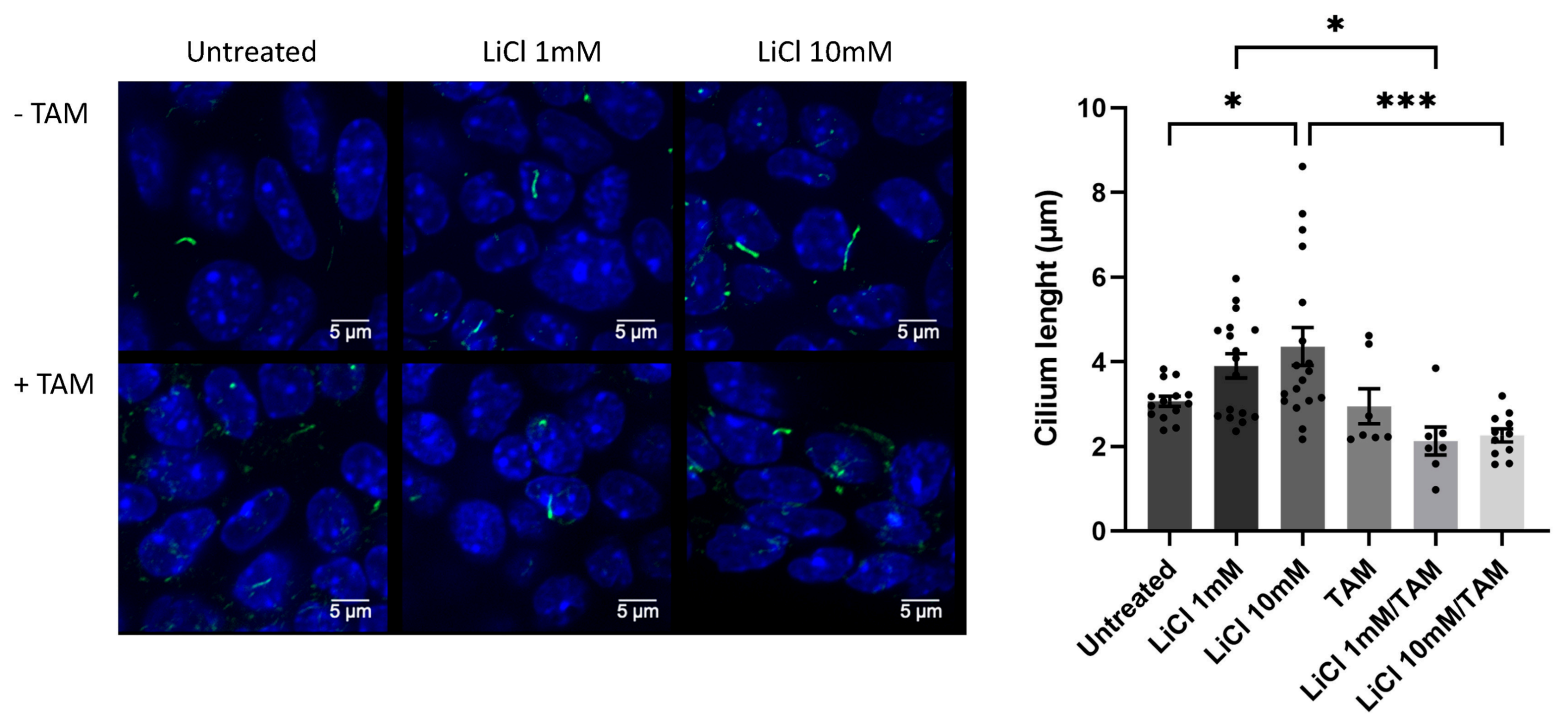
Effects of lithium on primary cilium elongation. MCD4 cells were treated as described in the Materials and Methods section, and confocal microscopy images were acquired. On the left, representative immunofluorescence images of the primary cilium in MCD4 cells. On the right, a bar plot shows the values of quantification of cilium length. Scale bar, 5 µm. Data are shown as means ± S.E.M. and analyzed by one-way ANOVA followed by Tukey’s multiple comparisons test (* *p* < 0.05 and *** *p* < 0.001).

Cilium formation and elongation are highly controlled processes that can be disrupted by lithium treatment in all cell types and organisms [25]. Confocal analysis in MCD4 cells revealed that treatment with lithium at 10 mM significantly increased the cilium length. A relevant but not significant increase in cilium length was also measured when cells were incubated with lithium at 1 mM. Interestingly, co-incubation with TAM abolished the lithium-induced cilium elongation (Figure 4). Data were analyzed using advanced NIS and the results are shown on the right panel of Figure 4 (Untreated: 3.07 ± 0.12; LiCl 1 mM: 3.9 ± 0.29; LiCl 10 mM: 4.36 ± 0.45; TAM: 2.95 ± 0.41; LiCl 1 mM/TAM: 2.13 ± 0.33; LiCl 10 mM/TAM: 2.26 ± 0.15; *n* = 3 independent experiments).

### 3.4. In Vitro Lithium Actions on EMT-like Changes

**Figure 5:**
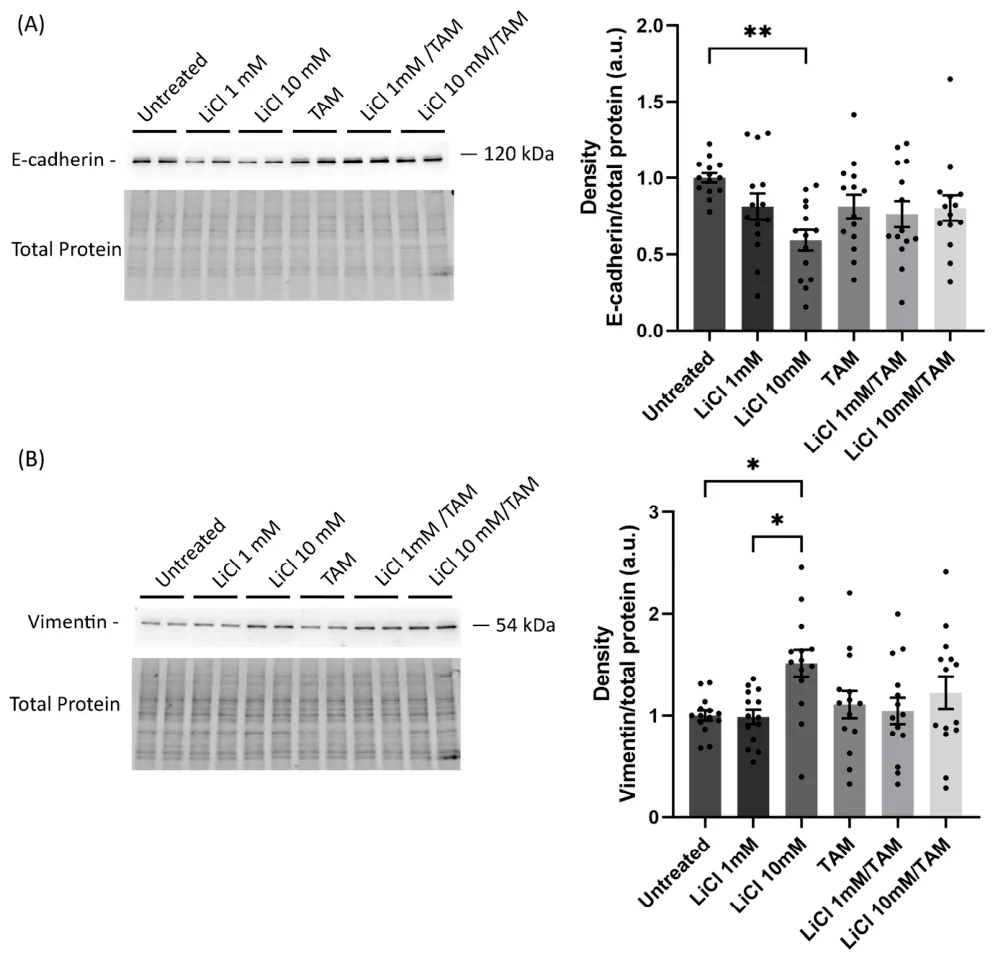
Effects of lithium on EMT markers expression in MCD4 cells. MCD4 cells were treated as described in the Materials and Methods section, and an equal amount (60 µg) of cell lysates was blotted and probed with the indicated antibodies. Immunodetection revealed a significant decrease in E-cadherin (**A**), and a significant increase in vimentin protein expression (**B**) in MCD4 cells incubated with lithium at 10 mM (LiCl 10 mM). Data are shown as means ± S.E.M. and analyzed by one-way ANOVA followed by Tukey’s multiple comparisons test (** *p* < 0.01 and * *p* < 0.05).

To investigate whether lithium promotes epithelial remodeling, the expression of the epithelial marker E-cadherin and the mesenchymal marker vimentin was evaluated by immunoblotting (Figure 5).

**Figure 6:**
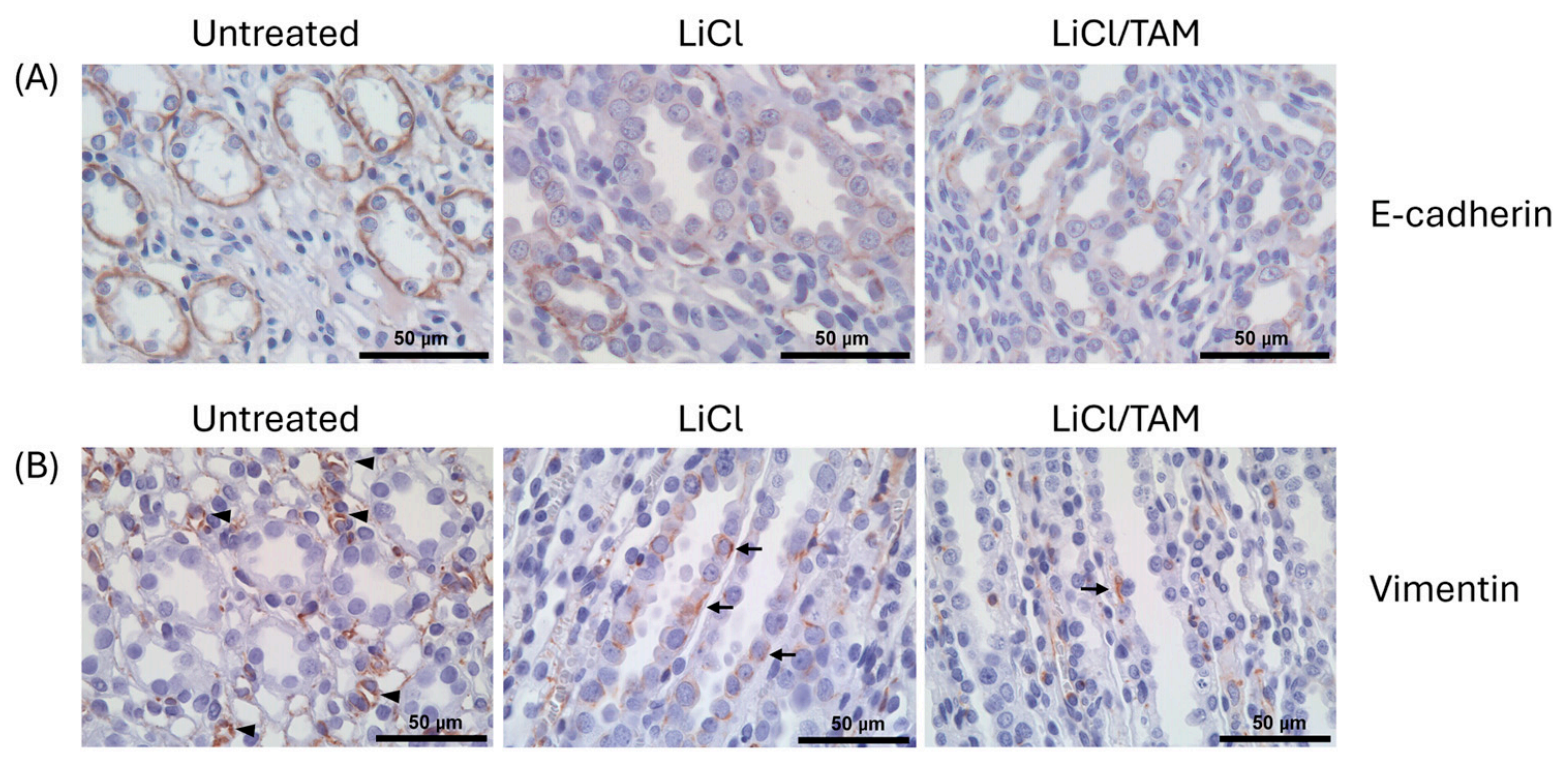
Effects of lithium on expression of EMT markers in kidney sections. Representative immunohistochemistry images of E-cadherin (**A**) and vimentin (**B**) expression in the proximal part of the inner medullary collecting ducts in untreated, Li-treated and Li/TAM-treated rats. Following lithium treatment, the majority of collecting ducts were either weakly labeled or negative for E-cadherin labeling, whereas only a few vimentin-positive cells were detected within the collecting ducts. Scale bar 50 µm.

Lithium treatment at 10 mM significantly reduced E-cadherin (LiCl 10 mM: 0.59 ± 0.07, *n* = 14) compared with untreated cells (Untreated: 1.00 ± 0.03, *n* = 14), while increasing vimentin (LiCl 10 mM: 1.51 ± 0.13, *n* = 14) protein abundance compared with both untreated (Untreated: 1.00 ± 0.05, *n* = 14) and 1 mM LiCl treated cells (LiCl 1 mM: 0.99 ± 0.07, *n* = 14). Tamoxifen alone did not significantly affect either E-cadherin expression or vimentin abundance. Notably, co-treatment with TAM partially prevented the lithium-induced reduction in E-cadherin levels (Figure 5A) (LiCl 10 mM = 0.59 ± 0.07, *n* = 14 vs. LiCl 10 mM/TAM = 0.80 ± 0.08,* n* = 14) and increase in vimentin expression (Figure 5B) (LiCl 10 mM = 1.51 ± 0.13, *n* = 14 vs. LiCl 10 mM/TAM = 1.22 ± 0.16, *n* = 14), thus restoring protein abundances to values comparable to those observed in untreated cells. These findings likely suggest that lithium induces changes consistent with a partial EMT-like phenotype or epithelial remodeling, characterized by reduced E-cadherin and increased vimentin expression. However, tamoxifen partially prevented these lithium-induced alterations, supporting its putative function in preserving epithelial homeostasis. Immunohistochemical staining for E-cadherin showed decreased labeling of basal and lateral plasma membrane domains in inner medullary collecting duct cells (IM-1) from lithium-treated rats compared with controls, confirming the loss of epithelial features induced by lithium exposure (Figure 6). The same was seen in the Li/TAM group. Furthermore, immunohistochemical analysis of kidney sections revealed the expression of some vimentin-positive cells within the inner medullary collecting ducts of lithium-treated rats (arrows). By contrast, vimentin staining was absent in collecting ducts from control animals. In controls, labeling was seen in blood vessels (arrowheads) and in glomeruli (not shown). Notably, only a few vimentin-positive cells were detected in Li/TAM-treated animals, supporting the ability of TAM to attenuate lithium-induced epithelial remodeling (Figure 6). To further investigate changes in EMT-like phenotype, the localization of β-catenin was evaluated as well (Figure S4). Interestingly, treatment with lithium significantly altered subcellular localization with a reduced membrane-associate labeling in collecting duct cells. However, TAM did not completely rescue the localization of β-catenin.

## 4. Discussion

The major findings of this study indicate that lithium treatment controls collecting duct epithelial remodeling through coordinated effects on ciliary length, cell fate pathways, and epithelial identity, while TAM partially preserves epithelial homeostasis and mitigates the lithium action on necroptosis-related markers. Lithium, used to treat psychiatric disorders, often promotes kidney damage and acquired nephrogenic diabetes insipidus [26]. These disturbances are mainly due to abnormal expression and function of different renal channels, including the epithelial sodium channel ENaC and renal water channels AQP2 and AQP3 [8,9]. However, more extensive studies show that the renal effects of lithium are not limited to altering water and sodium transport [27]. Lithium is also responsible for intracellular metabolic alterations [18], profound renal structural changes [28], and remodeling of the composition of renal tubular cells [14]. Indeed, lithium-induced tubular remodeling may ultimately lead to the formation of renal microcysts, which contribute to renal dysfunction by progressively occupying and disrupting functional renal tissue [29,30]. The development of renal cysts may result from uncontrolled cell proliferation and loss of epithelial polarity secondary to defects in primary cilium structure and function [31,32]. The length of the primary cilium is tightly regulated to preserve epithelial homeostasis and cellular signaling [33,34,35]. Lithium has been shown to disrupt this finely tuned process by inducing ciliary elongation, likely through alterations in intracellular trafficking and endocytosis mediated by dynamin and the Arp2/3 complex [25,36]. Considering the fundamental role of the primary cilium in coordinating cell proliferation, differentiation, and epithelial integrity [37], it is plausible to hypothesize that these alterations may be associated with the epithelial reorganization observed in lithium-induced nephrogenic diabetes insipidus. However, the alterations of primary cilium length may also represent a consequence of disrupted epithelial homeostasis induced by lithium. Consistent with this hypothesis, our findings showed that lithium was associated with epithelial remodeling, as evidenced by decreased E-cadherin and increased vimentin expression, suggesting the acquisition of selected features compatible with a partial epithelial-to-mesenchymal transition (EMT)-like phenotype. Importantly, these findings were further supported by immunohistochemical data, which show the appearance of vimentin-positive collecting duct cells in lithium-treated kidneys and the intracellular relocalization of β-catenin. Notably, TAM attenuates these lithium-induced alterations, indicating that its protective effects extend beyond regulating aquaporin expression [8,9] and possibly include the preservation of epithelial identity and function.

Importantly, the epithelial remodeling induced by lithium was accompanied by increased PCNA expression. Indeed, time-course studies in rats showed that in the early stage, lithium promotes the proliferation of principal cells followed by differentiation into proliferative intercalated cells [13,14]. Here, according to previous studies, lithium administration stimulated cell proliferation at both concentrations (1 and 10 mM) by promoting the proliferative marker, PCNA, in MDC4 cells. However, at lower lithium concentrations (1 mM), TAM rescued PCNA protein levels, whereas at the higher lithium levels (10 mM), proliferative signaling remained elevated despite TAM treatment. Therefore, the differential effect of TAM on lithium-induced PCNA expression may reflect the magnitude of the proliferative stimulus and suggest that actions of TAM, in terms of cell proliferation, are only partially effective when cells are exposed to higher lithium concentrations. Nevertheless, the higher PCNA expression under lithium treatment (10 mM) did not prevent TAM from restoring ciliary length, epithelial marker expression, and the expression of necroptosis-related proteins, indicating that increased proliferation and epithelial remodeling may represent partially independent processes. TAM may promote cell death by upregulating the expression and function of pro-apoptotic and necroptotic markers [38,39]. Of note, TAM caused a slight increase in the expression level of caspase-3. However, no relevant changes were found for the expression of the cleaved caspase-3 under all treatments, likely indicating that lithium and/or TAM did not induce cell apoptosis in this model. Exposure to 10 mM lithium was associated with a significant downregulation of the abundances of RIPK3 and MLKL, together with increased cell proliferation, primary cilium elongation, and EMT-like changes. These observations indicate that, as lithium exposure becomes more severe, a cellular program leading to epithelial remodeling might be induced. In addition, Western blotting analysis revealed that LC3-II levels remained unchanged by lithium regardless of TAM administration, suggesting that no substantial alteration of LC3-II was associated with the cellular responses observed in this cell model. Therefore, the observation that lithium induced epithelial remodeling without activation of apoptosis or autophagy prompted us to investigate whether alternative cell death pathways were involved. In this respect, it has been shown that the primary cilium downregulated the RIPK3-dependent necroptosis [40]. Necroptosis can be considered an alternative inflammatory programmed cell death, sharing similarities with apoptosis and necrosis [41], and requires the RIPK3-MLKL complex to form and activate [42]. Morphologically, necroptosis is similar to necrosis in terms of cellular swelling and collapse that leads to membrane permeabilization. Specifically, membrane rupture is operated by the consecutive activations of RIPK1-RIPK3, MLKL, and is followed by the release of intracellular molecules known as danger-associated molecular patterns (DAMPs) [43] that play a key role in tissue injury and inflammation [44]. Indeed, several studies proposed that lithium exerts anti-inflammatory actions by inhibiting fundamental pro-inflammatory molecules, including interleukins and the tumor necrosis factor-α (TNFα) [45], a known inducer of necroptosis. These observations lead us to speculate that lithium treatment might downgrade the abundance of necroptosis related proteins, thus possibly reducing cell necroptosis. Furthermore, TAM attenuates TNFα-dependent mitochondrial injury in heart disease and osteoporosis, suggesting multiple mechanisms by which TAM can exert beneficial effects. In line, previous studies have shown that TAM ameliorates lithium-induced polyuria and urinary concentration defects by restoring the expression of AQP2 and AQP3 [8,9]. We acknowledge that the lack of a direct assessment of phosphorylated MLKL (p-MLKL), a key marker of necroptosis, represents a limitation of our study, as its evaluation would have provided further insight into the potential involvement of this pathway in lithium-induced cellular alterations and the effects of tamoxifen treatment. Nevertheless, the primary aim of this study was not limited to the characterization of cell death mechanisms but rather to investigate the broader effects of lithium on collecting duct epithelial homeostasis beyond its established effects on aquaporin expression and function. Further studies are warranted to clarify the contribution of necroptosis to these processes. Our findings indicate that lithium profoundly alters collecting duct epithelial cell homeostasis, as evidenced by primary cilium elongation, altered proliferative activity, acquisition of a partial EMT-like phenotype, and downregulation of necroptosis-related proteins. Importantly, tamoxifen attenuated several of these lithium-induced alterations, supporting the concept that its protective effects extend beyond the restoration of AQP2 and AQP3 expression and include preservation of epithelial homeostasis. Collectively, these findings identify tamoxifen as a promising therapeutic strategy to mitigate the structural and functional alterations associated with lithium-induced nephrogenic diabetes insipidus.

## Data Availability

Data is contained within the article or supplementary material.

## Supplementary Materials

The following supporting information can be downloaded at: https://www.mdpi.com/article/10.3390/cells15211953/s1, Figure S1: Effects of lithium on proliferating cell nuclear antigen (PCNA) expression in MCD4 cells; Figure S2: Effects of lithium chloride on autophagy marker LC3A/B in MCD4 cells; Figure S3: Effects of lithium on apoptosis marker (Caspase-3 and cleaved Caspase-3) in MCD4 cells; Figure S4: Effects of lithium on expression and localization of β-catenin in kidney sections.

## Abbreviations

The following abbreviations are used in this manuscript:

TAM	Tamoxifen
Li	Lithium
EMT	Epithelial–Mesenchymal Transition
