# Supplementary material for: Beyond Aquaporins: Tamoxifen Preserves Collecting Duct Epithelial Homeostasis in Lithium-Induced Nephrogenic Diabetes Insipidus
Source: Cells. 2026 Oct 27;15(21):1953. doi: 10.3390/cells15211953 (PMC13761756; doi:10.3390/cells15211953)
Supplement: Supplementary file 1 [file cells-15-01953-s001.zip › cells-4501798-supplementary.pdf]

Article

Beyond Aquaporins: Tamoxifen Preserves Collecting Duct Epithelial Homeostasis in Lithium-Induced Nephrogenic Diabetes Insipidus

Mariangela Centrone [1,*], Ines Angelini [1], Mariagrazia D'Agostino [1], Annarita Di Mise [2], Barbara Barile [1], Grazia Paola Nicchia [1], Giampaolo Morciano [1], Stine Julie Tingskov [3], Xabier Sørtvedt [4], Birgitte Mønster Christensen [4], Marianna Ranieri [1], Giovanna Valenti [1], Rikke Nørregaard [3,5] and Grazia Tamma [1,*]

[1] Department of Biosciences Biotechnologies and Environment, University of Bari "Aldo Moro", 70125 Bari, Italy

[2] Department of Biotechnology and Biosciences, University of Milano-Bicocca, 20126 Milan, Italy
[3] Department of Clinical Medicine, Aarhus University, 8200 Aarhus N, Denmark

[4] Department of Biomedicine, Aarhus University, 8000 Aarhus C, Denmark

[5] Department of Renal Medicine, Aarhus University Hospital, 8200 Aarhus N, Denmark

* Correspondence: mariangela.centrone@uniba.it (M.C.); grazia.tamma@uniba.it (G.T.); Tel.: +39-080-5442388

SUPPLEMENTARY MATERIALS

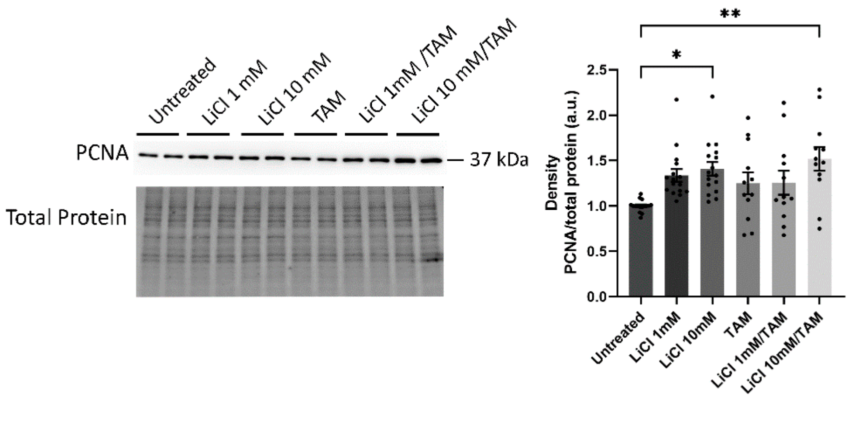

Figure S1 Effects of lithium on proliferating cell nuclear antigen (PCNA) expression in MCD4 cells. MCD4 cells were treated as described in the Materials and Methods section and an equal amount (60 µg) of cell lysates were blotted and probed with the indicated antibodies. Immunodetection revealed that PCNA protein expression is significantly increased in cells incubated with 10 mM lithium chloride. A significant increase is also observed when cells are incubated with 10 mM lithium chloride in the presence of TAM. Data are shown as means ± S.E.M. and analyzed by one-way ANOVA followed by Tukey's multiple comparisons test ($^{**}p<0.01$ and $^{*}p<0.05$).

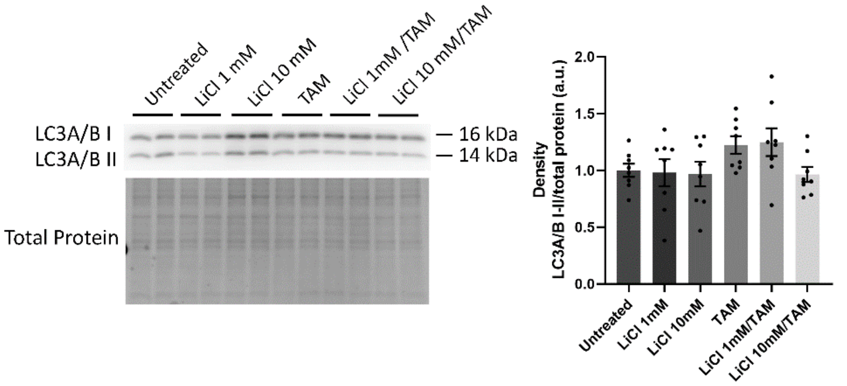

Figure S2 Effects of lithium chloride on autophagy marker LC3A/B in MCD4 cells. MCD4 cells were treated as described in the Materials and Methods section and an equal amount (60 μg) of cell lysates was blotted and probed with the indicated antibodies. No significant changes were observed in LC3A/B protein expression levels. Data are shown as means ± S.E.M. and analyzed by one-way ANOVA followed by Tukeys's multiple comparisons test.

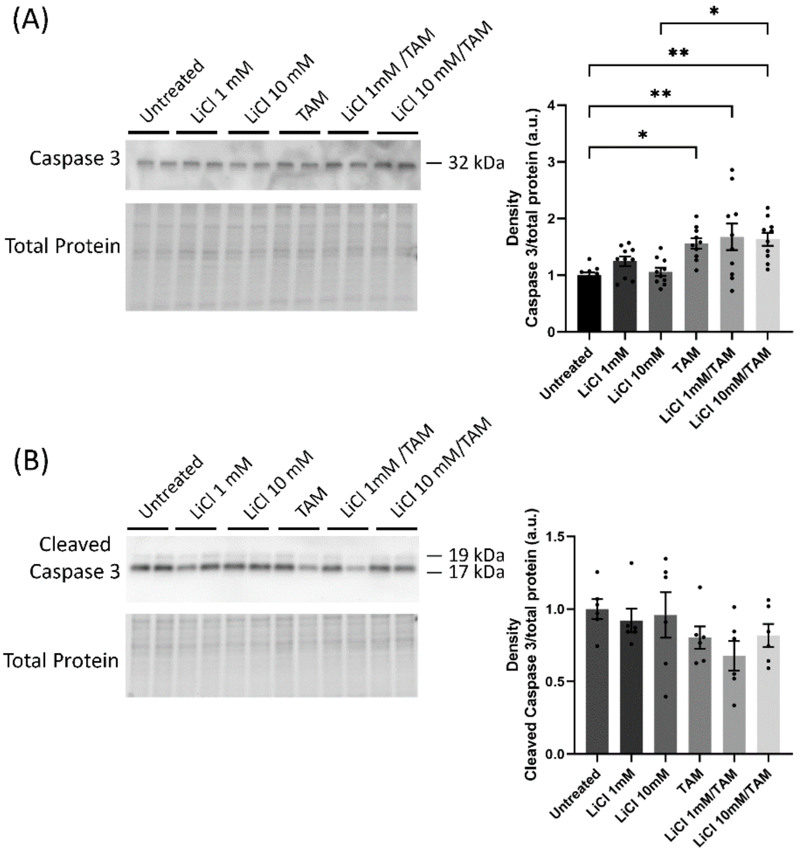

Figure S3 Effects of lithium on apoptosis marker (Caspase-3 and cleaved Caspase-3) in MCD4 cells. MCD4 cells were treated as described in the Materials and Methods section and an equal amount (60 μg) of cell lysates were blotted and probed with the indicated antibodies. Immunodetection revealed that Caspase-3 protein expression (A) was significantly increased in MCD4 cells incubated with TAM alone or in combination with lithium chloride (1 or 10 mM) compared with untreated cells. Whereas no significant changes were observed in cleaved Caspase-3 protein expression levels (B). Data are shown as means ± S.E.M. and analyzed by one-way ANOVA followed by Tukeys's multiple comparisons test (**p<0.01 and *p<0.05).

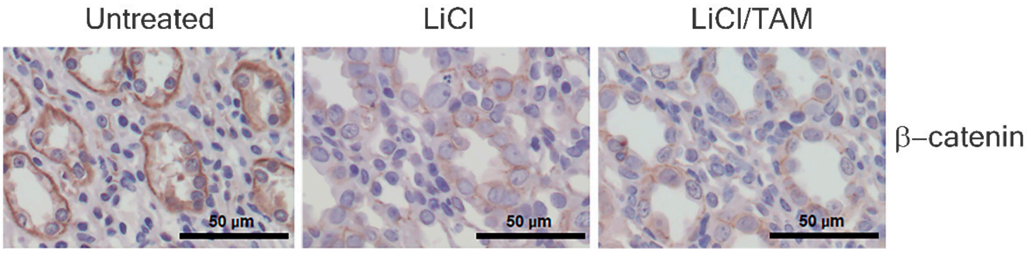

Figure S4. Effects of lithium on expression and localization of β-catenin in kidney sections. Representative immunohistochemistry images of β-catenin expression in the proximal part of the inner medullary collecting ducts in untreated, Li-treated and Li/TAM-treated rats. In controls kidney, β-catenin labeling was detected along the basal and lateral plasma membrane of collecting duct cells. Following lithium treatment, weaker labeling was observed along the lateral plasma membrane of collecting duct cells. A similar pattern was observed in Li/TAM-treated rats. Scale bar: 50 µm.
